# A Multicenter Assessment of the Outcomes and Toxicities of Foscarnet for Treatment of Acyclovir-Resistant Mucocutaneous Herpes Simplex in Immunocompromised Patients

**DOI:** 10.1093/ofid/ofae046

**Published:** 2024-02-05

**Authors:** Sarah P Hammond, Manickam Rangaraju, Melanie Sumner, Burkhard Timmler, Pranatharthi Chandrasekar, Robin K Avery

**Affiliations:** Divisions of Infectious Diseases and Hematology/Oncology, Massachusetts General Hospital, Dana-Farber Cancer Institute, Harvard Medical School, Boston, Massachusetts, USA; AiCuris Anti-infective Cures AG, Wuppertal, Germany; AiCuris Anti-infective Cures AG, Wuppertal, Germany; AiCuris Anti-infective Cures AG, Wuppertal, Germany; Karmanos Cancer Institute, Division of Infectious Diseases, Wayne State University School of Medicine, Detroit, Michigan, USA; Division of Infectious Diseases, Johns Hopkins University, Baltimore, Maryland, USA

**Keywords:** acyclovir, foscarnet, herpes simplex virus

## Abstract

**Background:**

Acyclovir-resistant mucocutaneous herpes simplex virus (HSV) infection is an uncommon problem typically seen in immunocompromised hosts. Systemic treatment options are limited. The performance of foscarnet and its toxicities in this population are poorly characterized.

**Methods:**

This was a multicenter retrospective study of adults treated with foscarnet for HSV infection between January 2012 and December 2017. Relevant data were collected including demographics, baseline conditions, previous anti-HSV medications, concomitant medications, HSV outcomes, and adverse events. Acyclovir-resistant HSV infection was defined based on genotypic or phenotypic testing results; refractory infection was defined as infection not improving after 5 days of treatment-dosed antiviral therapy in those not tested for resistance.

**Results:**

Twenty-nine patients had 31 episodes of HSV (15/18 resistant; among episodes without resistance testing, 7/10 refractory; 3 not evaluable) treated with foscarnet. All patients were immunocompromised including 19 (66%) with hematologic malignancy and 9 (31%) with HIV. Median duration of foscarnet was 16 days (range, 6–85 days). Fifteen episodes (48%) healed by the end of or after foscarnet. Median time to healing among those with resolution was 38 days (range, 9–1088 days). At least 1 adverse event during therapy was reported in 26 (84%) treatment episodes including 23 (74%) that were considered drug related. Common adverse events were electrolyte disturbance (20 [65%]) and kidney dysfunction (13 [42%]). Foscarnet was discontinued in 10 episodes (32%) due to an adverse event, including 6 due to kidney dysfunction.

**Conclusions:**

Among 31 episodes of HSV treated with foscarnet, only half resolved with treatment, and adverse events were common.

## Teaser

Acyclovir-resistant mucocutaneous herpes simplex virus (HSV) infection is a rare morbid illness typically treated with foscarnet. In this multicenter retrospective study of foscarnet therapy, half of 31 HSV episodes resolved during or after therapy; renal and electrolyte-related adverse events were common.

## Graphical abstract

**Graphical abstract:**
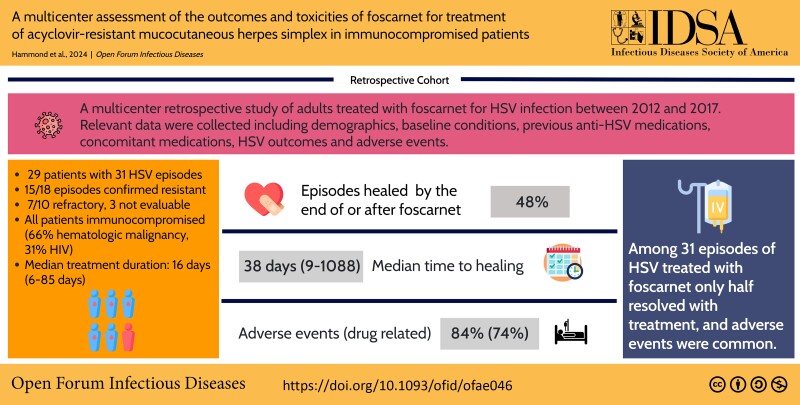
This graphical abstract is also available at Tidbit: https://tidbitapp.io/tidbits/a-multi-center-assessment-of-the-outcomes-and-toxicities-of-foscarnet-for-treatment-of-acyclovir-resistant-mucocutaneous-herpes-simplex-in-immunocompromised-patients

Herpes simplex virus (HSV) is a common cause of recurrent mucocutaneous and, less often, visceral infection. Immunocompromised individuals, including people with human immunodeficiency virus (PWH), transplant recipients, and patients with hematologic malignancy, are at particularly high risk for recurrent HSV infection. In 1 study, 70% of hematopoietic cell transplant (HCT) recipients developed HSV infection during transplantation in the absence of antiviral prophylaxis [1]. Acyclovir, a nucleoside analogue developed >40 years ago for the treatment of HSV and other herpesviruses, and the closely related prodrug valacyclovir, have been the mainstay of treatment and prevention of HSV infection and recurrence.

However, early in the development of acyclovir, before systemic acyclovir was commercially available, reports of resistance emerged in the immunocompromised population [2]. Acyclovir-resistant HSV infection is a rare clinical problem typically seen in the immunocompromised population, where it can cause painful protracted mucocutaneous disease associated with significant morbidity and mortality [3–5]. Acyclovir's antiviral activity hinges on initial phosphorylation by the HSV viral thymidine kinase enzyme; cellular kinases then phosphorylate the drug further and acyclovir-triphosphate then interferes with viral replication by inhibiting the viral polymerase. Thus, resistance is typically mediated through alterations in the viral thymidine kinase, but can also be mediated through alterations in the viral polymerase that can confer cross-resistance to other antiviral therapies.

Typically immunocompromised patients who develop acyclovir-resistant or refractory HSV infection have had previous repeated or prolonged exposure to acyclovir or other similar antiviral nucleoside analogues for prophylaxis or treatment of HSV [6]. Although historically PWH were at significant risk for resistant HSV infection, risk is seemingly lower in this patient population with contemporary antiretroviral therapy. In contrast, as novel immunosuppressive therapies for the management of hematologic malignancy grow, patients with hematologic malignancy remain a large at-risk population with substantial exposure to prophylactic anti-HSV antivirals. Acyclovir-refractory HSV infection is a clinical diagnosis that requires attention to host type, previous antiviral exposure, and response to standard acyclovir therapy. Definitive diagnosis of acyclovir-resistant HSV is made by phenotypic testing that can take several weeks to result [6]. Genotypic testing for acyclovir-resistant in clinical HSV isolates is not commercially available in the United States (US).

Systemic treatment options for acyclovir-resistant HSV infection are limited and include intravenous foscarnet, intravenous cidofovir, and high-dose or continuous infusion acyclovir [7, 8]. Foscarnet and cidofovir act by inhibiting the viral polymerase independent of the viral thymidine kinase, while continuous infusion acyclovir relies on the limited amount of initial acyclovir phosphorylation performed by cellular kinases in the absence of normal viral thymidine kinase activity [7].

All 3 systemic treatment options are associated with significant toxicities and require careful monitoring. Foscarnet, the treatment of choice for acyclovir-resistant HSV disease according to US guidelines [9], is given intravenously multiple times per day with concurrent hydration. Foscarnet carries a risk of kidney dysfunction and profound electrolyte disturbances including hypocalcemia that can lead to seizures. Less often, foscarnet can also cause noninfectious genital ulcers [10]. The need for frequent intravenous delivery and hydration, coupled with the electrolyte monitoring and repletion requirements of foscarnet, typically require hospitalization for initiation, which can be prolonged for some individuals. Some foscarnet toxicities are dose-dependent, so the fact that lower doses of foscarnet are needed to treat HSV than cytomegalovirus can mean the drug may be better tolerated in patients being treated for HSV infection. Systemic cidofovir is given intravenously intermittently, so this can obviate the need for initial hospitalization. However, cidofovir also carries a risk of nephrotoxicity, and less often uveitis. Because of its prolonged half-life, cidofovir cannot be cleared quickly when toxicity develops [11]. Last, continuous infusion acyclovir is infrequently chosen for treatment of acyclovir-resistant HSV, and requires careful monitoring given the potential for acyclovir to cause crystal nephropathy in patients who are not adequately hydrated.

To avoid the toxicities and other burdens of systemic therapy, compounded topical cidofovir and intralesional cidofovir are sometimes used for treatment of localized disease, though limited data suggest the topical approach alone may result in complete resolution as infrequently as 30% of the time [12]. More effective and less toxic oral options for the treatment of acyclovir-resistant HSV disease are needed.

Pritelivir is a novel oral antiviral agent in development for treatment of HSV infection that inhibits the viral helicase primase [13, 14]. A case series derived from a phase 2 study of this drug for treatment of mucocutaneous acyclovir-resistant HSV infection in immunocompromised patients showed that the drug was well tolerated, and 19 of 23 participants (83%) had complete resolution of HSV lesions by 28 days [15]. Treatment success with pritelivir given in an expanded access program has also been reported in several cases of resistant HSV infection [16–18]. A phase 3 study of pritelivir for treatment of mucocutaneous-resistant HSV in immunocompromised individuals is ongoing.

Literature describing the clinical outcomes and toxicities of treatment for resistant HSV infection with currently available therapy, particularly with the first-line choice, foscarnet, is lacking. Furthermore, currently available literature is largely limited to case reports and series that tend to describe treatment successes. Larger studies are needed to reduce this type of success bias. Last, understanding the utility of foscarnet for treatment of resistant HSV is important to help define treatment differences with novel therapies. We undertook this multicenter retrospective study in order to provide a more detailed description of the clinical outcomes and toxicities of foscarnet when used to treat resistant HSV infection.

## METHODS

This is a multicenter retrospective study in which each of 5 participating centers identified adult patients (aged ≥18 years) treated with foscarnet for HSV infection between 1 January 2012 and 31 December 2017. Subjects were identified by database search for foscarnet and HSV or relevant *International Classification of Diseases, Tenth Revision* codes or through microbiology record review for HSV isolates sent for resistance testing. Patients were included if they had a mucocutaneous HSV lesion that was accessible for visual examination for healing (including lesions visualized endoscopically). Patients were excluded if they had concurrent bacterial infection or other superinfection at the site of the HSV lesion or if they were pregnant, a prisoner, or had received foscarnet treatment as part of the phase 2 pritelivir clinical trial [15]. Patients with >1 discrete episode of HSV infection treated with foscarnet could contribute >1 episode of foscarnet treatment to the study. This study was approved by the institutional review board of each participating site.

Data collection included demographic information; medical history including details about immunocompromising conditions; anti-HSV medications taken before foscarnet; other medications taken up to 14 days before and concomitantly with foscarnet; physical examination findings including details about the size and location of HSV lesions; laboratory chemistry, hematology and virology parameters; and HSV disease and treatment parameters, including time to healing, presence and severity of lesional pain, and recurrence. Adverse events were defined as untoward medical occurrences during foscarnet therapy and were coded using the Medical Dictionary for Regulatory Activities, version 21.0. HSV infection was defined by cutaneous or mucosal findings clinically suggesting HSV infection, typically with microbiological confirmation of infection by HSV polymerase chain reaction, viral culture, or direct fluorescent antibodies to HSV.

Resistant HSV infection was defined by genotypic or phenotypic testing indicating acyclovir resistance. Refractory HSV infection was defined clinically as HSV infection with no improvement after at least 5 days of treatment doses of acyclovir, valacyclovir, ganciclovir, valganciclovir, or famciclovir antiviral therapy.

## RESULTS

Among the 5 participating sites, 29 patients were identified who were treated with foscarnet for HSV infection during the study period, including 2 who were treated with foscarnet for 2 separate episodes each. The clinical outcomes and toxicities associated with foscarnet treatment of HSV were studied in these 31 episodes of infection. Table 1 summarizes the demographic and baseline features of the 29 subjects. All subjects were immunocompromised, the majority of whom had a hematologic malignancy. No patient in the cohort had received a solid organ transplant, and 9 (31%) were PWH. Among the 19 patients (66%) with hematologic malignancy, 16 had undergone allogeneic HCT and 1 had undergone autologous HCT. Among the 16 allogeneic HCT recipients, 10 had graft-versus-host disease. Table 2 shows episode-level details, including underlying conditions and previous steroid treatment.

**Table 1.** Baseline Characteristics Among 29 Patients With 31 Episodes of Herpes Simplex Virus Treated With Foscarnet

Characteristic	No. (%) of Patients
Male sex	19 (66)
Age, y, median (range)	54 (34–74)
Immunocompromising condition	
Hematologic malignancy^a^	19 (66)
HIV infection	9 (31)
Other^b^	1 (3)
Hypogammaglobulinemia	7 (24)
Corticosteroids^c^	15 (49)

Abbreviation: HIV, human immunodeficiency virus.

^a^Acute leukemia/myelodysplastic syndrome/myeloproliferative disorder (n = 9), lymphoma or myeloma (n = 7), chronic leukemia (n = 3).

^b^Thymoma and Good syndrome.

^c^Among the 31 episodes of herpes simplex virus (HSV) in 29 participants, 15 were on systemic corticosteroids before the HSV episode.

**Table 2.** Episode-Level Characteristics of 31 Episodes of Herpes Simplex Virus Treated With Foscarnet

Episode	Underlying Condition	Corticosteroids Within 30 d (Indication)	HSV Site	HSV Type	Acyclovir Resistance Testing	Anti-HSV Therapy Within 30 d	Duration Foscarnet, d	Reason Foscarnet Stopped	Time to Resolve, d	Recurrence
1	HIV	None	Anal, sacral	2	Resistant	Valacyclovir	85	Healed	85	Yes
2	NHL, allo-HCT	None	Anal, oral	2	Resistant	Valacyclovir	44	AE		
3	HIV	None	Suprapubic	2	Resistant	None	22	Improved		
4	CLL, allo-HCT	Yes (GVHD)	Anal, limb, torso, oral	1	Resistant	Ganciclovir, valacyclovir	40	AE		
5	HIV	None	Genital	2	Resistant	Valacyclovir	34	Healed	34	Yes
6	Good syndrome/thymoma	None	Anal, limb, torso, genital	2	Resistant	Acyclovir	45	Other^a^	1088	No
7	CTCL, allo-HCT	Yes (CTCL)	Genital	2	NA	Acyclovir	11	Healed	12	No
8	HIV	None	Oral	ND	NA	None	48	Healed	48	No
9	HIV	NA	Anal, genital	2	Resistant	NA	15	Improved		
10	HIV	NA	Genital	2	NA	NA	15	Improved		
11	HIV	NA	Genital	ND	NA	NA	14	Improved		
12	HIV	NA	Genital	ND	NA	NA	10	Improved		
13	MDS, allo-HCT	Yes (ES)	Genital	2	Resistant	Ganciclovir, topical acyclovir	16	Healed	16	Yes
14	MM, autologous HCT	Yes (MM)	Oral	1	Resistant	Famciclovir, valacyclovir/acyclovir	16	Improved	37	No
15	NHL, CLL, allo-HCT	Yes (GVHD PPx, mucositis)	Oral	1	NA	Acyclovir	17	Healed	17	No
16	NHL, CLL, allo-HCT	Yes (GVHD PPx)	Oral	1	NA	Acyclovir, ganciclovir	11	Died of other cause		
17	Myelofibrosis, allo-HCT	Yes (GVHD PPx)	Anal	2	Resistant	Acyclovir, topical cidofovir	13	AE		
18	ALL, allo-HCT	Yes (mucositis)	Oral	1	Susceptible	Acyclovir	46	AE		
19	CLL, allo-HCT	None	Oral	1	Resistant	Valacyclovir	15	Healed	15	Yes
20	NHL	Yes (NHL)	Oral, anal	1 + 2	Resistant	None	8	AE		
21	MDS, allo-HCT	None	Oral	1	NA	Valacyclovir	14	Failure		
22	ALL, allo-HCT	Yes (GVHD)	Oral	1	NA	Valacyclovir	50	AE		
23	ALL, allo-HCT	Yes (GVHD)	Oral	1	NA	Valacyclovir	6	Improved	38	No
24	ALL, allo-HCT	Yes (GVHD)	Oral	1	NA	None	10	AE		
25	CLL	None	Oral	1	Resistant	Valacyclovir/acyclovir	12	Improved	43	Yes
26	HIV	None	Genital	2	Susceptible	Valacyclovir/acyclovir, valganciclovir/ganciclovir	22	Improved		
27	NHL, allo-HCT	Yes (radiation pneumonitis)	Anal	1	Resistant	Valacyclovir, topical cidofovir	60	AE		
28	ALL, allo-HCT	Yes (GVHD)	Oral	1	Resistant	Valacyclovir/acyclovir	14	Improved	40	Yes
29	AML, NHL, allo-HCT	None	Oral	1	NA	Valacyclovir	10	Healed	9	Yes
30	CLL, allo-HCT	Yes (GVHD)	Oral	1	Susceptible	Brincidofovir	21	AE	26	No
31	AML, NHL, allo-HCT	None	Oral	1	NA	Valacyclovir	22	Healed	22	Yes

Abbreviations: AE, adverse event; ALL, acute lymphoblastic leukemia; allo-HCT, hematopoietic cell transplant; AML, acute myelogenous leukemia; CLL, chronic lymphocytic leukemia; CTCL, cutaneous T-cell lymphoma; ES, engraftment syndrome; GVHD, graft-versus-host disease; HCT, hematopoietic cell transplant; HIV, human immunodeficiency virus; HSV, herpes simplex virus; MDS, myelodysplastic syndrome; MM, multiple myeloma; NA, not available; ND, not determined; NHL, non-Hodgkin lymphoma; PPx, prophylaxis.

^a^Patient ran out of medication.

Table 3 summarizes the HSV infection characteristics for the 31 episodes treated with foscarnet. There were slightly more HSV episodes due to HSV type 1 than HSV type 2, and there were more episodes exclusively involving oral mucosa than other sites or multiple sites. Most patients had been treated with acyclovir, valacyclovir, or a closely related anti-HSV drug, and a minority had been treated with oral brincidofovir or concurrent topical cidofovir within 30 days before foscarnet was started for HSV therapy. Most episodes were due to resistant or refractory HSV; of the 18 HSV episodes where genotypic or phenotypic resistance testing was performed, 15 (83%) were confirmed as acyclovir resistant. Of the remaining 13 episodes where genotypic and phenotypic testing was not performed, refractory status could be determined in 10 episodes, of which 7 were refractory (70%). The initial size of the HSV lesion was recorded in only 9 of 31 episodes. The median size reported was 81.0 mm^2^, but there was a wide range (4–4900 mm^2^).

**Table 3.** Herpes Simplex Virus (HSV) Characteristics and Treatment History Among 29 Patients With 31 Episodes of HSV Treated With Foscarnet

Characteristic	No. (%) (N = 31 Episodes)
HSV type	
HSV-1^a^	18 (58)
HSV-2^a^	14 (45)
Site of active HSV infection	
Oral	15 (48)
Anogenital	12 (39)
Oral and anogenital^a^	3 (10)
Other^b^	1 (3)
Prior systemic anti-HSV treatment (n = 27)^c^	
Acyclovir, valacyclovir, or famciclovir	21 (78)
Ganciclovir or valganciclovir	4 (15)
Brincidofovir	1 (4)
Prior local anti-HSV treatment (n = 27)^d^	
Topical acyclovir	1 (4)
Topical cidofovir	2 (7)
Acyclovir refractory or resistant (n = 28)^e^	22 (79)
Genotypically or phenotypically defined acyclovir resistance (n = 18)^f^	15 (83)

Abbreviation: HSV, herpes simplex virus.

^a^One episode of foscarnet treatment was used for concurrent acyclovir-resistant oral HSV-1 and acyclovir-resistant anogenital HSV-2.

^b^Includes an episode of suprapubic HSV vegetans.

^c^Includes antivirals given within 30 days of foscarnet therapy in the 27 episodes for which prior antiviral therapy was available; 3 patients treated with acyclovir/valacyclovir were also treated with other agents before foscarnet.

^d^Includes topical therapy given within 30 days of foscarnet therapy including 27 episodes for which prior and concomitant antiviral therapy was available; all patients who were treated with local therapy also received acyclovir, valacyclovir, or ganciclovir also before foscarnet therapy.

^e^Includes 18 who had virologic phenotypic or genotypic testing performed for acyclovir resistance and 10 who were evaluable for refractory status.

^f^Eighteen of 31 episodes had virologic phenotypic or genotypic testing performed for acyclovir resistance; 3 were not resistant.

Of the 31 episodes of HSV that were treated with foscarnet, 15 (48%) were reported to heal by the end of or after foscarnet therapy (Table 4). The median duration of foscarnet therapy was 16 days (range, 6–85 days). The median time to lesion healing was 38 days (range, 9–1088 days), which included healing even after foscarnet was stopped. Seven episodes (23%) healed within 28 days. Among the 16 episodes not reported as healing, 14 (45%) did not resolve after foscarnet therapy and information was not available after foscarnet therapy on 2 episodes. Among the 15 episodes that resolved, 8 (53%) of these recurred at a median of 229 days (range, 31–1030 days). Assessment of pain related to the HSV infection was reported in 17 of 31 episodes (55%). The number of days of pain relative to the total duration of the episode in days per patient ranged from 2% to 100% with a median of 100% and a mean of 88% in the group where pain data were available. Pain cessation was reported in 5 of 17 episodes at a median on 74 days.

**Table 4.** Foscarnet Treatment Details and Outcomes

Treatment/Outcome Detail	N = 31 Episodes
Duration of foscarnet, d, median (range)	16 (6–85)
HSV lesion healing^a^	
Complete resolution	15 (48)
No resolution	14 (45)
Time to healing, d, median (range) (n = 15)	38 (9–1088)^b^
Time to recurrence after healing, d, median (range) (n = 8)	229 (31–1030)

Abbreviation: HSV, herpes simplex virus.

^a^Data on resolution were not available for 2 episodes.

^b^Includes healing after stopping foscarnet treatment.

At least 1 adverse event occurring during foscarnet therapy was reported in 26 of 31 (84%) episodes including 23 episodes (74%), during which at least 1 adverse event was considered drug related. Table 5 shows the most commonly reported adverse events by organ system reported in 20% or more of the 31 episodes. The most frequent adverse events were related to electrolyte disturbance and kidney function and a majority of these were attributed to foscarnet therapy. Foscarnet was discontinued in 10 episodes (32%) due to an adverse event including in 6 episodes (19%) due to kidney disorders. Foscarnet was not discontinued due to electrolyte disturbances, but electrolyte adverse events attributed to foscarnet occurred in 19 of 31 (61%) HSV episodes including hypocalcemia and hypophosphatemia, which both occurred in 11 of 31 episodes (35%). No episodes of seizure were reported.

**Table 5.** The Most Common Adverse Events Associated With Foscarnet Therapy by Organ System (N = 31 Episodes)

Organ System	Any Adverse Event	Foscarnet-Related Adverse Events	Adverse Event Leading to Foscarnet Discontinuation
Infection	9 (29)	…	…
Pneumonia	5 (16)	…	…
Staphylococcal bacteremia	3 (10)	…	…
Electrolyte disorders	20 (65)	19 (61)	…
Hypocalcemia	11 (35)	11 (35)	…
Hypokalemia	9 (29)	8 (26)	…
Hypomagnesemia	5 (16)	5 (16)	…
Hypophosphatemia	11 (35)	11 (35)	…
Renal and urinary disorders	13 (42)	9 (29)	6 (19)
Kidney injury^a^	13 (42)	9 (29)	6 (19)
Respiratory disorders	9 (29)	…	…
Hypoxia	2 (6)	…	…
Respiratory distress	2 (6)	…	…
Respiratory failure	3 (10)	…	…
Tachypnea	3 (10)	…	…

Data are presented as No. (%).

^a^Kidney injury includes the Medical Dictionary for Regulatory Activities classifications: acute kidney injury, renal impairment, increased blood creatinine, azotemia, and worsening of chronic kidney disease.

There were 11 episodes (35%) during which at least 1 serious adverse event occurred, most of which were due to infections or respiratory disorders. Two episodes of HSV that were complicated by serious adverse events were due to acute kidney injury, 1 of which led to foscarnet discontinuation. Nine study subjects had 13 adverse events leading to death, none of which were considered to be related to foscarnet. Most adverse events leading to death were due to disorders of the respiratory system or infection.

## DISCUSSION

Acyclovir-resistant HSV is a rare but formidable clinical problem that tends to affect profoundly immunocompromised patients. Resistant HSV lesions can be painful, large, multifocal, and prolonged, leading to substantial patient discomfort. Unfortunately, for decades the primary systemic treatment options—including foscarnet, cidofovir, and continuous infusion acyclovir—have remained static, and have substantial toxicities as well as suboptimal efficacy. Additionally, treatment outcomes for resistant HSV with these regimens remain understudied, with descriptions often limited to case reports or case series that are impacted by success bias.

Foscarnet is the preferred treatment choice for resistant HSV, but as demonstrated in this cohort, it is limited in efficacy and is associated with substantial renal and electrolyte toxicities. Among 31 episodes of HSV infection treated in this immunocompromised cohort, fewer than half resolved fully by the end of foscarnet therapy or in follow-up afterward. Adverse events due to electrolyte disturbance were present in more than half of the episodes, while adverse events related to kidney disorders occurred in more than one-third of patients, leading to treatment discontinuation in 19% of episodes.

Adverse events leading to death occurred in 9 subjects, none of which were judged to be related to foscarnet therapy, but highlight the vulnerability of this patient population [3, 4]. Immunocompromised patients with acyclovir-resistant HSV infection tend to be fragile and medically complex with high baseline morbidity. Toxic antiviral therapies like foscarnet or the other alternative therapies in this population can cause adverse events such as acute kidney injury that can synergize with toxicities related to other common concomitant medications such as tacrolimus and trimethoprim-sulfamethoxazole. Furthermore, foscarnet therapy given over adequate durations to treat resistant HSV typically requires careful management in the inpatient setting that can lead to prolonged hospitalization. The cumulative effect of both antiviral and concomitant medication toxicities, coupled with deconditioning resulting from prolonged hospitalization, can make foscarnet therapy difficult to recover from in this morbid patient population. Monitoring of renal function and electrolytes at least 2–3 times a week, vigorous hydration, and electrolyte repletion are necessary and require clinicians to be vigilant, especially when foscarnet is administered in the outpatient setting. Even with the utmost care, harmful toxicities can occur. Less toxic and more effective therapies are needed to improve outcomes in this challenging rare illness.

This study was limited due to the small study size and the constraints of retrospective data collection, particularly across multiple medical centers. Lesion measurements and pain scales were not consistently serially recorded, as these would be in the context of a clinical trial. We were unable to capture some information of interest such as occurrence and magnitude of HSV viremia, changes in HSV shedding, and changes in the size of lesions over the course of treatment. We were also not able to collect retrospective data describing common clinically observed potential downstream effects of foscarnet therapy such as volume overload and substantial peripheral edema resulting from the large volume of fluid and electrolyte repletion typically given with the drug.

In summary, this cohort of 29 immunocompromised individuals treated with foscarnet for 31 episodes of HSV demonstrated the limited efficacy and substantial toxicity of foscarnet therapy, and the unmet need for a less toxic, orally bioavailable therapy for resistant/refractory HSV infection.

## Notes

***Author contributions.*** M. R., M. S., and B. T. designed the study. P. C., R. K. A., and S. P. H. collected data. S. P. H. drafted the manuscript. All authors critically reviewed and edited the manuscript.

***Patient consent.*** This study was approved by the institutional review board of each participating site. Individual patient consent was not required due to the retrospective noninterventional nature of the study.

***Financial support*.** This work was funded by AiCuris Anti-infective Cures AG.
